# Beneficial Effects of Trypsin Inhibitors Derived from a Spider Venom Peptide in L-Arginine-Induced Severe Acute Pancreatitis in Mice

**DOI:** 10.1371/journal.pone.0061049

**Published:** 2013-04-15

**Authors:** Weiwen Ning, Yongjun Wang, Fan Zhang, Hengyun Wang, Fan Wang, Xiaojuan Wang, Huaxin Tang, Songping Liang, Xiaoliu Shi, Zhonghua Liu

**Affiliations:** 1 College of Life Sciences, Hunan Normal University, Changsha, Hunan, China; 2 Department of Digestion, the Second Xiangya Hospital, Central South University, Changsha, Hunan, China; Klinikum rechts der Isar der TU München, Germany

## Abstract

HWTI is a 55-residue protein isolated from the venom of the spider *Ornithoctonus huwena*. It is a potent trypsin inhibitor and a moderate voltage-gated potassium channel blocker. Here, we designed and expressed two HWTI mutants, HWTI-mut1 and HWTI-mut2, in which the potassium channel inhibitory activity was reduced while the trypsin inhibitory activity of the wild type form (approximately 5 EPU/mg) was retained. Animal studies showed that these mutants were less toxic than HWTI. The effects of HWTI and HWTI-mut1 were examined in a mouse model of acute pancreatitis induced by intraperitoneal injection of a large dose of L-arginine (4 mg/kg, twice). Serum amylase and serum lipase activities were assessed, and pathological sections of the pancreas were examined. Treatment with HWTI and HWTI-mut1 significantly reduced serum amylase and lipase levels in a dose dependent manner. Compared with the control group, at 4 mg/kg, HWTI significantly reduced serum amylase level by 47% and serum lipase level by 73%, while HWTI-mut1 significantly reduced serum amylase level by 59% and serum lipase level by 72%. Moreover, HWTI and HWTI-mut1 effectively protected the pancreas from acinar cell damage and inflammatory cell infiltration. The trypsin inhibitory potency and lower neurotoxicity of HWTI-mut1 suggest that it could potentially be developed as a drug for the treatment of acute pancreatitis with few side effects.

## Introduction

Severe acute pancreatitis (SAP) is an inflammatory process of the pancreas that causes acinar death, local complications or even multiple organ failure. The hospital mortality rate associated with severe acute pancreatitis is very high (20%–30%) despite the availability of advanced treatment modalities for this disease. Although the precise pathogenic mechanism of severe acute pancreatitis remains unclear, the generally accepted theory is that it is initiated by the intra-pancreatic activation of proteases. The activation of pancreatic enzymes results in the digestion of local acinar cells, which induces the release of more enzymes, triggering local and systemic inflammatory responses [1]–[4].

The activation of trypsinogen plays a key role in the progress of SAP [5]. Trypsin is produced in the pancreas as an inactive proenzyme, trypsinogen, a small proportion of which is activated in acinar cells and then inhibited by the pancreatic secretory trypsin inhibitor (PSTI). In parallel, the protease activated receptor-2 (PAR-2), located on the surface of acinar and duct cells, mediates a negative feedback loop that inhibits pancreatic trypsin secretion when the extracellular concentration of trypsin increases [6]. An excessive activation of trypsinogen to trypsin may disturb this balance and contribute to the development of pancreatitis. Activated trypsin may lead to cell damage, which can in turn trigger the release of more trypsin. Trypsin activates kallikrein, a serine protease that liberates kinins such as bradykinin and kallidin from their precursor kininogens. These kinins can increase vascular permeability and induce vasodilatation and neutrophil accumulation. Moreover, the release of trypsin and other toxic factors into the systemic circulation can lead to cardiovascular and pulmonary collapse [6].

Given the critical role of trypsin in the pathogenesis of SAP, protease inhibitors have been considered as a potential treatment for SAP. Indeed, the earliest clinical application of trypsin inhibitors for the treatment of acute pancreatitis was reported in the 1960s [7],and in the 1980s, Japanese researchers demonstrated the safety and effectiveness of trypsin inhibitors for the treatment of acute pancreatitis [7]. Recent studies have shown that trypsin inhibitors suppress vascular smooth muscle contraction by inhibiting calcium influx [8], increase blood flow to the pancreas, protect vascular endothelial cells from damage by oxygen radicals [9], and effectively reduce the release of inflammation factors [10]. However, the clinical efficacy of antiproteases is still a matter of controversy. The use of aprotinin, a broad spectrum Kunitz type protease inhibitor with strong action against trypsin, chymotrypsin and kallikrein, for the treatment of acute pancreatitis has been studied for the last 50 years with disappointing results [7]. However, some researchers argue that most of the studies addressing the use of aprotinin for the treatment of acute pancreatitis have not been conducted adequately, have inappropriate end-points, and most importantly, have not attained adequate plasma and peritoneal levels of aprotinin to produce sufficient inhibitory activity. These researchers proposed that a well-powered study with adequate aprotinin dosing may clarify its clinical benefit in severe acute pancreatitis [7].

HWTI is a bifunctional protein that possesses inhibitory activity against trypsin and blocks voltage-gated potassium channels. It was isolated from the venom of the spider *Ornithoctonus huwena*. It consists of 55 amino acids with 6 cysteine residues forming 3 pairs of disulfide bonds, and belongs to the BPTI/Kunitz-type serine protease inhibitor family. HWTI is a very strong trypsin inhibitor with an inhibitory potential approximately 30-fold higher than that of aprotinin. However, unlike aprotinin, HWTI is also a moderate blocker of voltage-gated potassium channels including Kv1.1, 1.2 and 1.3. A structure-function relationship study indicated that the binding sites corresponding to the two functions of HWTI are independent from each other on the surface of the protein [11]. Therefore, it is possible to reduce the inhibitory action of HWTI on potassium channels by mutating specific residues without affecting the trypsin inhibitory activity of the protein. Moreover, because HWTI is a stronger trypsin inhibitor than aprotinin, the mutated analogues could be developed as potential agents for the treatment of SAP with less neurotoxicity. In this study, two mutants of HWTI were designed and expressed using a yeast expression system. The protective effect of the recombinant proteins against SAP was further evaluated in a mouse SAP model induced by intraperitoneal injection of a large dose of L-arginine (L-Arg).

## Materials and Methods

### Materials

Escherichia coli strain TOP10 was used for production of plasmids. Saccharomyces cerevisiae strain S78 (Leu2, Ura3, Rep4) and vector pVT102U/α were used for expression of HWTI and its mutants. Uracil-deficient YSD and YPD media were used for propagation of yeast transformants. Enzymes used for DNA manipulation were purchased from MBI Fermentas (USA). The gel extraction mini kit was from QIAGEN. A DNA ladder was purchased from Takara. Tryptone and yeast extract were from Oxoid. Proteose peptone was obtained from Sangon (Shanghai, China), and the carrier DNA was from Clontech. All other reagents were from Sigma, and all chemicals were of analytical reagent grade. Male Wistar rats weighing 220 to 250 g were used. Kunming mice (of both sexes, each half) with a body weight of 20±2 g were used for assessing toxicity and pharmacodynamics. All animal experiments were approved by the Animal Welfare Committee of Hunan Normal University.

### Primer Design and Construction of Plasmids

The cDNA sequence of HWTI was obtained by the RACE method. PCR primers were designed based on the sequence of HWTI [11], [12] and synthesized by GenScrip. HWTI-forward (5′- CGTCTAGATAAGAGAATAGATACATGCCGTTTGCCC-3′) was used as sense primer with an XbaI restriction site, HWTI-reverse (5′- CCGAAGCTTTTATGCTTTTGCACATCTTTTC-3′) was used as antisense primer with a HindIII restriction site. The amplified products were cloned into the expression plasmid pVT102U/α after being digested with XbaI and HindIII. PCR primers corresponding to HWTI-mut1 (forward: 5′-CGTCTAGATAAGAGAATAGATACATGCCTTGAACCCTCTGACACTGGGAG-3′, reverse: 5′-CCGAAGCTTATGCTTTTGCACATCTTTTCATG-3′) and those for HWTI-mut2 (forward: 5′-CGTCTAGATAAGAGAATAGATACATGCCTTGAACCC-3′, reverse: 5′-CCGAAGCTTATGCTTTTGCACATCTTTTCATG -3′) were designed according to the sequence of pVT102U/α-HWTI and synthesized by GenScrip. The amplified target fragments were obtained by inverse-PCR using pVT102U/α-HWTI-R25A as a template. Conditions for PCR were as follows: 94°C for 5 min; 9°C for 1 min; 63°C for 1 min. 72°C for 1 min, 27 cycles, and 72°C for 1 min. Reverse-PCR was used to amplify the target fragment from the HWTI primer [13].

### Expression of Recombinant HWTI and its Mutants

Plasmids confirmed by DNA sequencing were transformed into S. cerevisiae S78 by using the LiCl method [13]. One positive transformant on the YSD plate was cultured in 25 mL of YSD liquid media for 24 hours and then transferred to 750 ml of YSD liquid media at a dilution of 1∶30 for 3–4 days of cultivation. Fermentation liquor was mixed with 1 M NaAc (pH = 4.2) to reach a final concentration of NaAc of 0.1 M. The mixture was centrifuged at 11,000 rpm for 20 min, and the supernatant was collected for further purification.

### Purification of Recombinant HWTI and its Mutants

The culture supernatant was filtered through a 0.45 µM filter membrane and applied onto a CM-Sepharose column (3×30 cm) previously equilibrated with 0.1 M NaAc. After the column was balanced with 0.1 M NaAc with 10 column volumes, the chromatography was developed by a stepwise elution with increasing concentrations of NaCl (0.1, 0.2, 0.5 and 1 M) in 0.1 M NaAc. The recombinant HWTI and its mutants were eluted at 0.5 M NaCl. The corresponding fractions were collected, lyophilized and desalted by reverse-phase HPLC (RP-HPLC). The molecular weights of HWTI and its mutants were determined by MALDI-TOF MS (Bruker, UltrafleXtreme MALDI-TOF-TOF).

### Analysis of the Trypsin Inhibitory Activities of HWTI and its Mutants

Trypsin inhibitory activity was assessed using two methods. The first method is Dixon method [14]. Our previous study showed that HWTI is a competitive inhibitor of trypsin [11]. The equation (1) governing this relationship is given below:
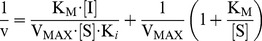
(1)


Plots were prepared of the reciprocal of rate of metabolite formation (1/*v*) versus inhibitor concentration [I] at each substrate concentration [S]. Velocity (*v*) was measured according to the method of Erlanger et al [15] as modified by Benjakul et al [16] using Nα-Benzoyl-DL-arginine 4-nitroanilide Hydrochloride (BAPNA) as a substrate. Inhibitors (HWTI, HWTI-mut1 or HWTI-mut2) with appropriate dilutions in 100 µL buffer solution and 10 µL 0.6 mg/mL trypsin (10 µL buffer for control) were added to 96-well plates. According to our preliminary tests, the concentrations of HWTI were 25, 50, 75, 100 and 200 nM, while the concentrations of the two mutants were 2.5, 5.0, 7.5, 10 and 20 nM. The inhibitor and trypsin mixture was preincubated for 15 min at 30°C, and then 30 µL substrate (the final concentration was 0.2 mM or 0.5 mM) was added into the mixture. After incubation for 25 min at 30°C, 10 µL of 60% acetic acid (v/v) was added to terminate the reaction. The absorbance of reaction mixture was read at 410 nm and *v* was then calculated using the following formula (2):
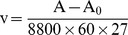
(2)where 8800 (M^−1^×cm^−1^) is the coefficient of p-nitroaniline; *A* and *A_0_* are absorbance at 410 nm of the sample and the control, respectively. The resulting straight lines were analyzed by linear fitting Sigmaplot. Estimates of Ki were obtained by simultaneously solving two sets of equations sequentially. The X axis value of intersection of these pairs of lines represents the value of -Ki.

The second method is an acid-base titration method described in European Pharmacopoeia 5.0 [17]. The inhibition activity of HWTI and the mutants were determined by measuring its inhibitory action on a solution of trypsin of known activity. Briefly, titration with 0.1 M NaOH neutralized the acid released by trypsin hydrolyzing the substrate (benzoylarginine ethyl ester hydrochloride) to maintain the solution at pH 8.0±0.1. The temperature of the solution was maintained at 25±0.1°C, the reaction was continued for 6 min and the volume of NaOH added was recorded every 1 min. The dose of the trypsin in test group was two-fold of that in control group, and our experiments indicated that 0.6 mg/mL of an inhibitor (HWTI, HWTI-mut1 or HWTI-mut2) could just inhibit approximate half of trypsin in test group, that is, the residual trypsin activity of the test group was similar with that in control because the titration curves of the three proteins were observed to approximately overlap with that of control group. The numbers of millilitres of 0.1 M NaOH used per second in the test group (n_2_ mL) and in the control group (n_1_ mL) were determined, respectively, and therefore the inhibitory activity in European Pharmacopoeia Units (EPU) per milligram was calculated from the expression (3):
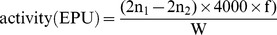
(3)where 4000 is coefficient, f is calibration factor of NaOH (f was 0.217 in this study), and W is weight of an inhibitor (mg).

### Potassium Channel Inhibitory Activity of HWTI and its Mutants

Potassium channel activity was assessed on rat dorsal ganglia (DRG) neurons using the whole cell patch clamp technique. Rat DRG neurons were rapidly dissociated and maintained in a short-term primary culture and inhibition of potassium channels on rat DRG neurons was examined according to the method described by Yuan et al. [11]. Experimental data were acquired and analyzed by the program Pulsefit 8.0 (HEKA). All data are presented as means ± standard error.

### Intracerebroventricular (i.c.v) Injection

96 mice(48 males and 48 females)weighing 18-22 g were randomly divided into 12 groups:control, HWTI (110, 165, 250, 375 and 562.5 µg/kg), HWTI-mut1 (110, 165, 250, 375, 562.5 and 25,000 µg/kg). Mice were anesthetized with ethyl ether before injection. A 5 mm×5 mm incision was made at the midpoint between ears and the cranium was exposed. On one side of the intersection of sagittal sutures and lambdoidal sutures, a hole was made with a micro injector and 15 µL of solution was injected into the 4th ventricle. The animal’s behaviors were observed for 48 hours after the injection [18], [19].

### Evaluation of Therapeutic Effects in a Mouse SAP Model

Mouse SAP was induced by intraperitoneal injection of a large dose of L-arginine (Arg) as described previously [18], [19]. Briefly, 130 male mice weighing 22-26 g were fed with a standard diet and housed in a standard shoebox cage at 20–25°C with a 12 hours dark/light cycle. A solution of L-Arg (8%) was prepared in normal saline and the pH was adjusted to 7.0. The solution was administered intraperitoneally at a dose of 4 g/kg. 1 hour after the first injection, another injection was executed at the same dose. Animals were then returned to the cages and allowed free access to food and water. 6 hours after the first injection, animals were randomly divided into 12 groups: 0.25 mg/kg (×2), 0.5 mg/kg(×2), 1 mg/kg(×2), 2 mg/kg(×2), 4 mg/kg(×2), normal and control. HWTI and HWTI-mut1 were dissolved in normal saline and diluted to the required concentrations (0.25–4 mg/kg). A volume of 0.2 mL of HWTI or HWTI-mut1 solutions of different concentrations was administered intraperitoneally 8, 24 and 48 hours after the first injection of L-Arg. The control group received a sham injection of saline. 72 hours after the injection of L-Arg, blood was collected by using retro-orbital bleeding method, and centrifuged at 3,000 rpm for 10 min at 4°C.

The serum obtained was used for measurement of amylase and lipase activity. The serum amylase activity was measured using starch-iodine method described by Caraway [20], [21], [22]. Briefly, 10 µL serum sample (H_2_O for control) was incubated with 500 µL reagent 1 (starch) for 7.5 min at 37°C, 500 µL reagent 2 (iodine), 2.5 mL H_2_O were immediately added to the reaction solution and the amount of color development was determined by measuring the absorbance at 660 nm. The serum amylase activity was determined by the difference of absorbance between control and sample. The serum lipase activity was measured using turbidimetric assay described by Shihabi ZK et al [23]. 25 µL sample (saline for control) and 2 mL substrate buffer were mixed and the absorbance of the mixture was measured at 420 nm 30 seconds later. Thereafter, the mixture was incubated in 37°C for 10 min and measured at 420 nm. The serum lipase activity was determined by difference between first and second absorbance.

The pancreas was rapidly removed en bloc, fixed in formalin, embedded in paraffin, sectioned, stained with hematoxylin (H) and eosin (E), and evaluated by a pathologist unaware of the sample identity. 3–5 randomly selected pancreas sections were scored for pancreas edema, inflammatory cell infiltration, pancreas hemorrhage and necrosis according to quantitative method described in the references [24]–[26]. The scoring criterion for edema and inflammatory cell infiltration: 0, absent; 1, mild; 2, moderate; 3, severe. The scoring criterion for hemorrhage: 0, absent; 3, less than 2 foci; 5, 3–5 foci; 7, more than 5 foci. The scoring criterion for necrosis: 0, absent; 3, less than 5%; 5, 5−20%; 7, more than 20%. The sum of the scores represented severity of overall pancreatic damage.

### Statistical Analysis

Data are expressed as means ± SE. Data analysis was done by applying unpaired two-tailed Student’s t-test with significance being assigned to P values<0.05.

## Results

### Expression and Purification of Recombinant HWTI and its Mutants

**Figure 1:**
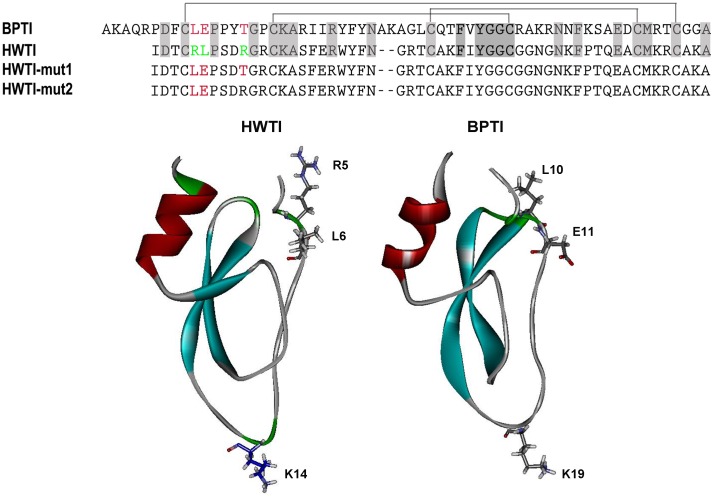
Structure and sequence comparison of HWTI and BPTI. (A) Amino acid sequences of HWTI and BPTI and the two mutants. The disulfide bonds are indicated above the sequences. Gray shading indicates identical residues between HWTI and BPTI. The potassium channel inhibition-related residues in HWTI are labeled in green, while the corresponding residues in BPTI are labeled in red. In the two BPTI mutants, the mutated residues are indicated in red. (B) 3D structures of HWTI (PDB code, 2JOT) and BPTI (PDB code, 1LD5). The residues corresponding to the inhibitory activity against trypsin and potassium channels are indicated.

HWTI and aprotinin are Kunitz-type serine protease inhibitors. They share an identical disulfide linkage pattern and therefore have the same structural motif, namely the Kunitz type motif (Fig. 1). However, unlike aprotinin, HWTI can also block voltage-gated potassium channels in addition to its trypsin inhibitory activity. In our previous study [11], we demonstrated that both Arg5 and Leu6 are key residues for HWTI binding to potassium channels, whereas Lys14, which is located in a side chain protruding from the molecular surface, plays a critical role in the trypsin inhibitory function of HWTI. These active sites are in separate and independent locations on the surface of HWTI (Fig. 1), indicating that the substitution of one active site should not affect the activity of the other. Aprotinin, which is able to inhibit trypsin but not potassium channels, shares the key residue Lys19 for trypsin inhibition with HWTI, while Arg5 and Leu6 in HWTI correspond to Leu10 and Glu11 in aprotinin (Fig. 1). This suggests that the potassium channel inhibitory activity of HWTI could be reduced by changing Arg5 and Leu6 to Leu5 and Glu6, respectively. In addition, our previous study showed that replacing Arg10 by Thr enhanced the trypsin inhibitory activity of HWTI [11]. We therefore constructed two mutants with the aim of generating a stronger trypsin inhibitor with lower potassium channel inhibition activity. Arg5, Leu6 and Arg10 were replaced by Leu5, Glu6 and The10, respectively, generating HWTI-mut1, while in HWTI-mut2, only Arg5 and Leu6 were replaced by Leu5 and Glu6, respectively.

**Figure 2:**
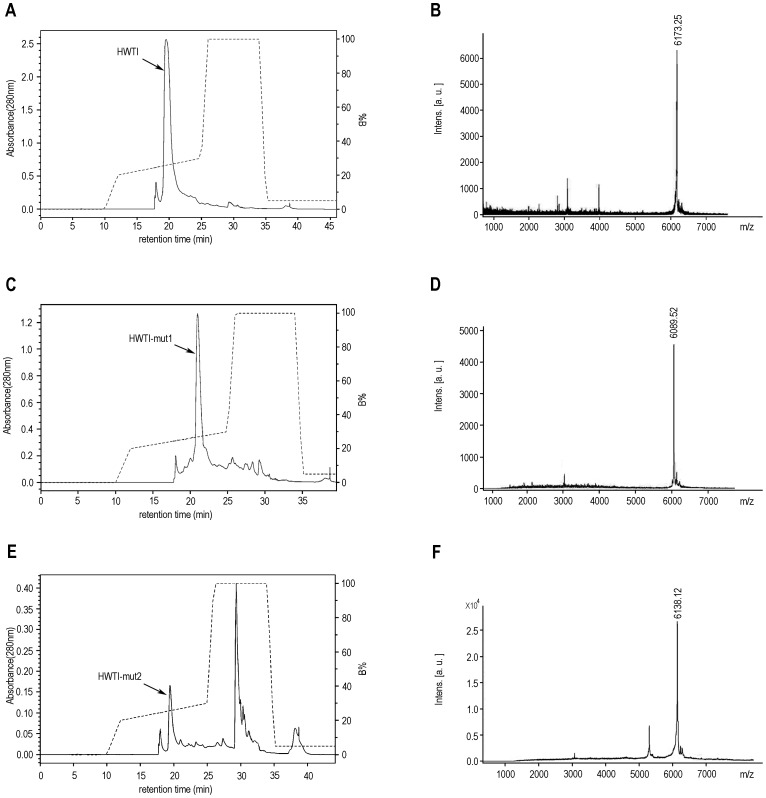
Purification of HWTI and the two mutants. Fractions collected from the CM-Sepharose column were applied to a RP-HPLC column equilibrated with 0.1% trifluoroacetic acid for further purification at a flow rate of 2 mL/min (buffer B, 0.1% trifluoroacetic acid in acetonitrile) with an increase from 20% to 30% over 13 minutes. The molecular mass of the purified protein was measured by MALDI-TOF mass spectrometry. The retention time of HWTI was 19.23 min. (A), and the measured MW was 6,173.25 (B). For HWTI-mut1, the retention time was 21 min (C), and the measured MW was 6,089.52 (D). The retention time of HWTI-mut2 was 19.5 min (E), and the measured MW was 6,138.12 (F).

The recombinant HWTI and its mutants were expressed by using the vector pVT102U in *S. cerevisiae* strain S78. The expression vectors (pVT102U/α-HWTI, -HWTI-mut1 and -HWTI-mut2) were constructed and confirmed by DNA sequencing, and then transformed into *S. cerevisiae* S78. After the yeast were cultured for 3 days in YPD media, the recombinant HWTI, HWTI-mut1 and HWTI-mut2 were recovered in the supernatant, as determined by SDS-PAGE. The supernatant was first subjected to cationic chromatography and the fraction eluted by 0.5 M NaCl contained the target product as determined by MALDI-TOF MS. This fraction was further purified by RP-HPLC. As shown in Fig. 2, the retention time of the target product was approximately 20 min. The measured monoisotopic masses of HWTI, HWTI-mut1, and HWTI-mut2 were 6,173.25, 6,089.52, and 6,138.12 Da, respectively (Fig. 2), which is consistent with the masses estimated from their sequences. The yields of the three recombinant proteins were 9.3 mg/L for HWTI, 6.7 mg/L for HWTI-mut1, and 2.1 mg/L for HWTI-mut2. The yield of HWTI-mut2 was much lower than the yields of HWTI and HWTI-mut1.

### Trypsin Inhibitory Activity of HWTI and its Mutants

**Figure 3:**
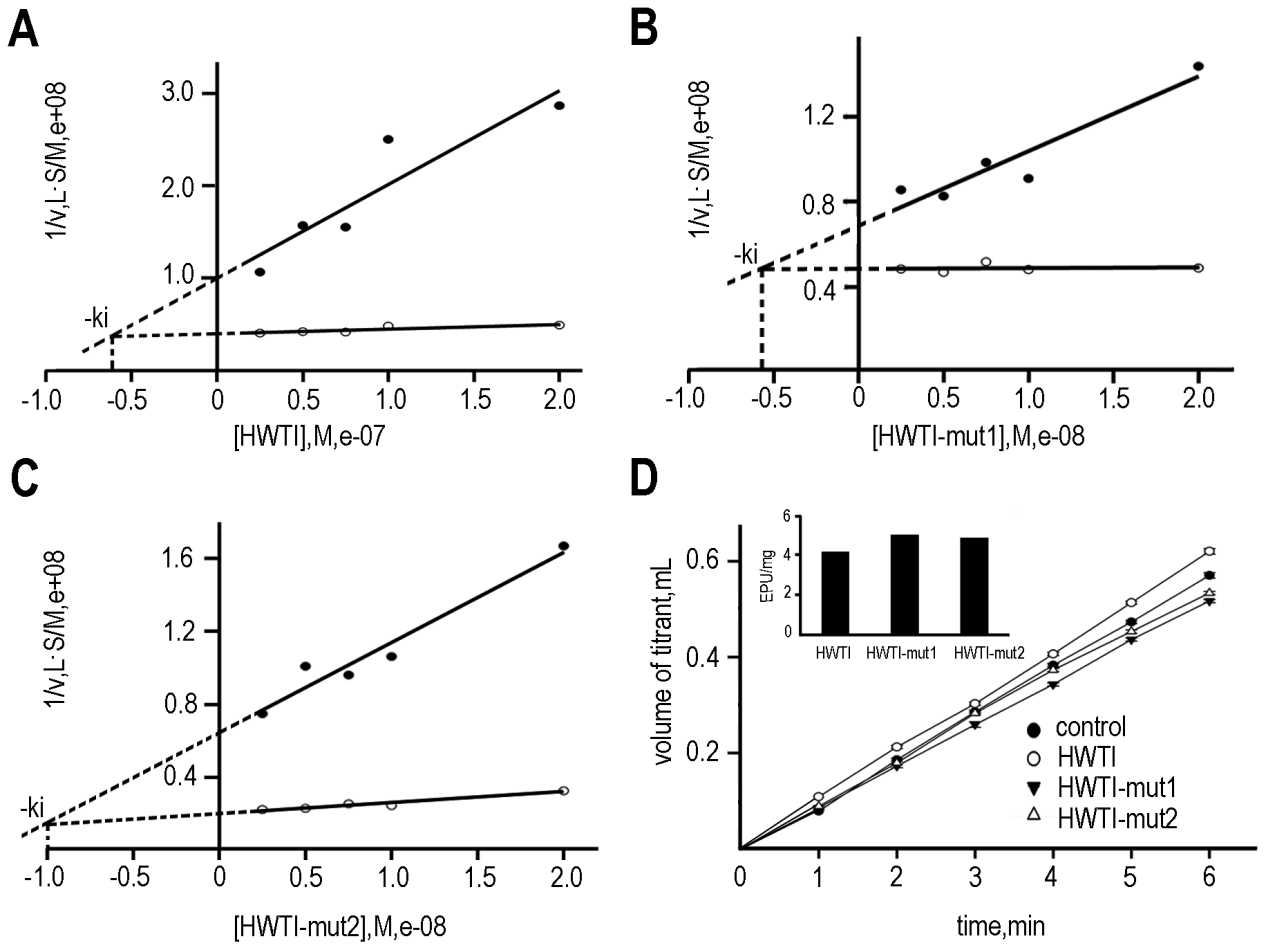
Effect of HWTI and the two mutants on trypsin. Estimation of Ki of HWTI (A), HWTI-mut1 (B) and HWTI-mut2 (C) by Dixon plots. Y axis represents reciprocal of velocity (1/*v*) and X axis represents concentrations of HWTI/HWTI-mut1/HWTI-mut2. The concentration of substrate was 0.2 mM (open circle) or 0.5 mM (closed circle). (D) The titration curves of HWTI/HWTI-mut1/HWTI-mut2. The concentration of HWTI and the two mutants was 0.6 mg/mL. Each measurement was conducted in triplicate. Data are expressed as mean±SE (n = 3). *Inset,* showing the trypsin inhibitory activity (expressed in EPU/mg) of the three proteins.

The trypsin inhibitory activities of the recombinant proteins were assessed by spectrophotometry and titration based methods. In the spectrophotometry method, inhibitory constants (Ki) of HWTI and the two mutants were determined by using Dixon plots in order to compare their inhibitory abilities on trypsin. As shown in Fig. 3, Ki values of the three proteins were 6.3e−8 M for HWTI (Fig. 3A), 5.9e−9 M for HWTI-mut1 (Fig. 3B) and 1.0e−8 M for HWTI-mut2 (Fig. 3C). HWTI-mut1 showed slightly higher trypsin inhibitory activity than HWTI and HWTI-mut2. Next, the trypsin inhibitory activities of the recombinant proteins were further determined by using a titration method that was previously described for aprotinin [17]. In this method, inhibitory activity is calculated from the difference between the initial and residual activity of trypsin and expressed as European Pharmacopoeia Units (EPU). One EPU inhibits 50 percent of the enzymatic activity of 2 microkatals of trypsin. Fig. 3D shows the titration curves of the three proteins, from which the volume (n_1_) of 0.1 M NaOH used per second in the test group was determined to be 1.72e−3 mL for HWTI, 1.44e−3 mL for HWTI-mut1, or 1.48e−3 mL for HWTI-mut2; the volume (n_2_) of 0.1 M NaOH used per second in the control group was 1.58e−3 mL. Therefore, the trypsin inhibitory activities were calculated to be 4.18 EPU/mg for HWTI, 5.01 EPU/mg for HWTI-mut1 and 4.88 EPU/mg for HWTI-mut2 (Fig. 3D, *inset*). Again, the inhibitory ability of HWTI-mut1 was slightly higher than those of HWTI and HWTI-mut2. Taken together, our results indicate that mutation of the residues in the potassium binding site of HWTI has no effect on its trypsin inhibitory activity. Moreover, a more potent trypsin inhibitor was successfully designed and expressed.

### Comparison of the Potassium Channel Inhibitory Activities of HWTI and the Two Mutants

**Figure 4:**
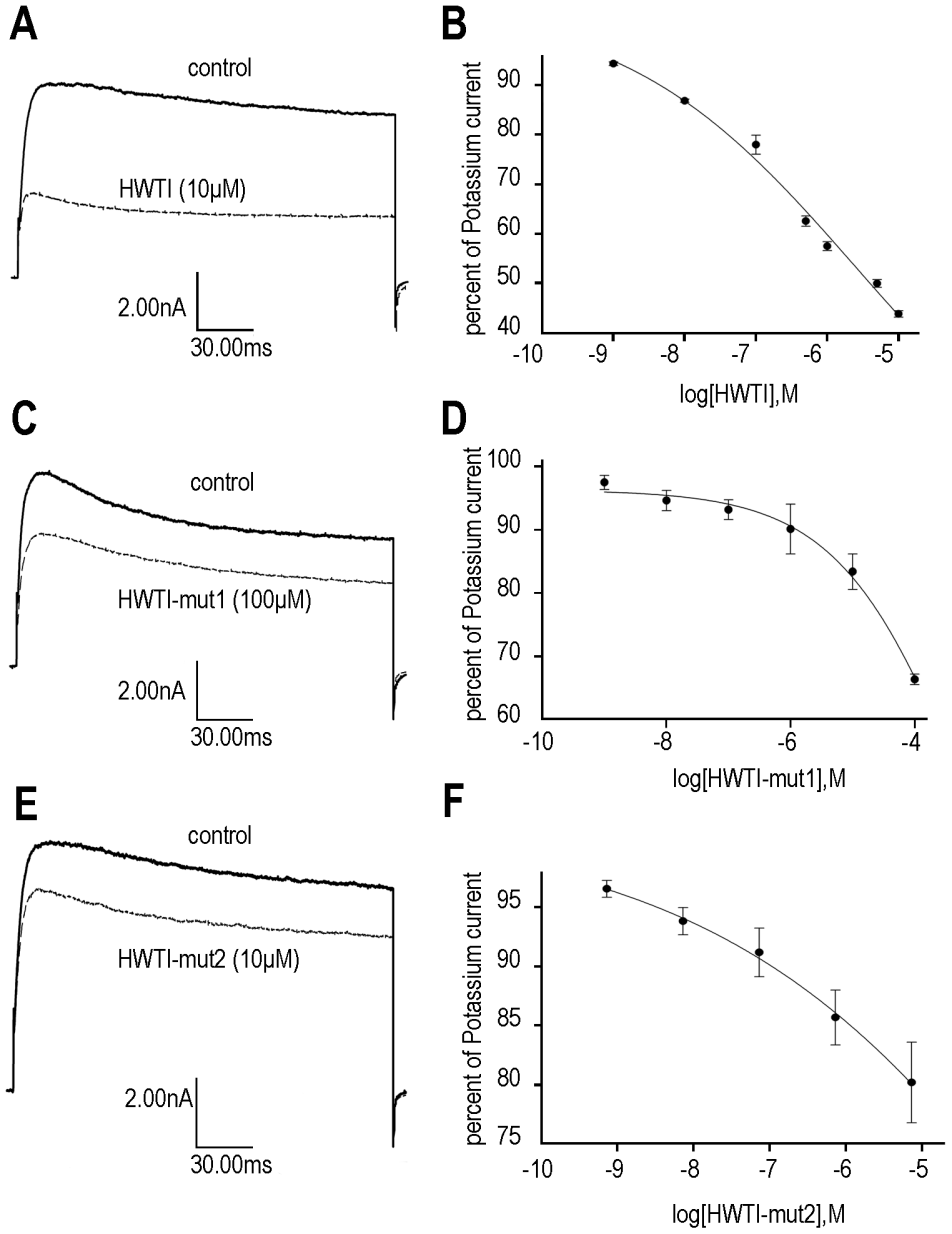
Effect of HWTI and the two mutants on potassium channel activity in rat DRG cells. Potassium currents were recorded by whole-cell patch-clamp with rapidly dissociated DRG neurons. (A) Currents were recorded before and after the application of 10 µM HWTI. (B) Currents measured in the presence of HWTI were converted into percent of control currents and plotted versus the log[HWTI], with an IC_50_ of 3.92 µM. (C) Currents were recorded before and after the application of 10 µM HWTI-mut1. (D) Currents measured in the presence of HWTI-mut1 were converted into percent of control currents and plotted versus the log[HWTI-mut1]. (E) Currents were recorded before and after the application of 10 µM HWTI-mut2. (F) Currents measured in the presence of HWTI-mut2 were converted into percent of control currents and plotted versus the log[HWTI-mut2].

The potassium channel inhibitory activities of the three proteins were measured in rat DRG neurons by using the whole-cell patch technique [19]. As shown in Fig. 4, the three proteins inhibited potassium channels in rat DRG neurons in a dose-dependent manner, but the inhibitory activities of HWTI-mut1 and HWTI-mut2 were much lower than that of HWTI. Consistent with our previous study, approximately 60% of the total potassium current was inhibited by HWTI at the maximal concentration of 10 µM (Fig. 4A), and the IC_50_ value was 3.92 µM (Fig. 4B). However, HWTI-mut1 or HWTI-mut2 at a dose of 10 µM inhibited less than 20% of the total potassium currents (Fig. 4C and 4E). Because the percent inhibition by the mutant proteins was below 50% even at the maximal concentration of 10 µM, their IC_50_ values could not be determined (Fig. 4D and 4F). However, the potassium channel inhibitory activities of both mutants were considerably lower than that of HWTI. These results confirmed that we successfully designed and expressed trypsin inhibitors with reduced potassium channel inhibitory activity.

The neurotoxic properties of the recombinant proteins were examined by intracerebroventicular injection into mice. One minute after injection of HWTI, mice showed signs of hysteria or panic as observed in frantic behavior and flipping. Within 48 hours, HWTI even resulted in 27.5% to 75% death of mice with the increase of doses (110−562.5 µg/kg) and the median lethal dose (LD_50_) was calculated to be 217.15 µg/kg by using the modified Kaber method. On the contrary, HWTI-mut1 showed considerably lower neurotoxicity than HWTI, as determined by the absence of the symptoms described for HWTI even after injection with a maximal dose of 25 mg/kg HWTI-mut1 (40-fold higher than the maximal dose of HWTI) and no death of mice were observed. These results indicated that the mutations introduced successfully reduced the neurotoxicity of HWTI.

### Protective Effect of HWTI and HWTI-mut1 in a Mouse Model of SAP

Intraperitoneal injection of a large dose of L-arginine is known to induce SAP in mice. This method was first established by Mizunuma et al. in 1984 and later improved to evaluate treatments for SAP [27]–[29]. In the present study, we applied this method to examine the protective effect of HWTI and HWTI-mut1. Mice became sluggish and sleepy after the administration of the first dose of L-Arg (4 g/kg in saline), and the symptoms became more apparent after the second dose. Most studies rely on serum amylase and lipase levels, two diagnostic indexes for acute pancreatitis, and acinar cell injury to assess the severity of pancreatitis. As compared with the normal group, SAP mice showed higher serum amylase and lipase levels and increased acinar cell damage.

**Figure 5:**
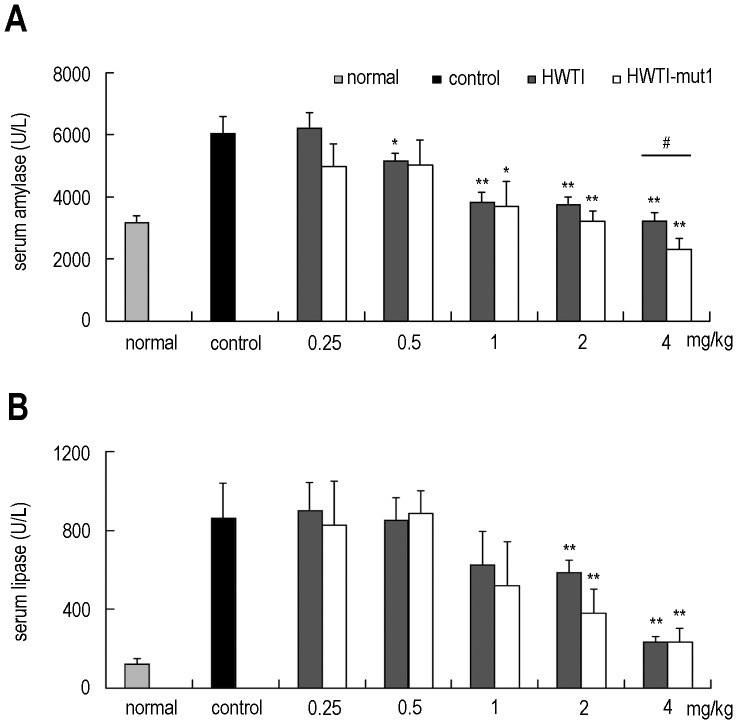
Effect of HWTI and HWTI-mut1 on the reduction of serum amylase and lipase levels. Severe acute pancreatitis in mice was induced by injecting 4 g/kg L-arginine (8%) twice with a 1 hour interval. HWTI and HWTI-mut1 were administered intraperitoneally 8, 24, and 48 hours after the first injection of L-arginine. Mice were sacrificed at 72 hours and serum was collected for the measurement of amylase and lipase levels. (A) HWTI and HWTI-mut1 significantly reduced serum amylase levels at a dose of 1 mg/kg. At the maximal dose (4 mg/kg), the amylase level was nearly half of that of the control group. HWTI-mut1 was more effective at reducing amylase levels in the 4 mg/kg group than HWTI. (B) HWTI and HWTI-mut1 significantly reduced serum lipase levels at a dose of 2 mg/kg. In the 4 mg/kg group, serum lipase level was nearly 1/3 of that of the control group. Data are shown in IU/L and expressed as mean+SE (n = 8–12). * denotes p<0.05 compared with the control group; ** denotes p<0.01 compared with the control group; ^#^ denotes p<0.05 between lined groups.

Injection of L-Arg significantly increased serum amylase levels from 3,177 U/L to 6,144 U/L (Fig. 5A). Treatment of these mice with HWTI and HWTI-mut1 decreased the serum amylase levels in a dose dependent manner. For HWTI, a significant decrease of serum amylase level was observed at a HWTI dose of 0.5 mg/kg (5,187 U/L, p<0.05). Serum amylase levels continued to decrease at a dose of 1 mg/kg (3,849 U/L, p<0.01) HWTI and reached values comparable to those of the normal group at the maximum dose of 4 mg/kg (3,243 U/L, p<0.01). The same dose-effect relationship was observed after treatment with HWTI-mut1. A significant decrease of serum amylase level was observed with HWTI-mut1 at a dose of 1 mg/kg (3,688 U/L, p<0.05), and the serum amylase level was 2,316 U/L (p<0.01) at 4 mg/kg HWTI. In contrast to HWTI, HWTI-mut1 had no obvious effect at 0.5 mg/kg, but was more effective in reducing the serum amylase level at 4 mg/kg (p<0.05).

The experimental induction of pancreatitis caused a significant rise in serum lipase compared to the normal group (from 128 U/L to 886 U/L) (Fig. 5B), and treatment with HWTI and HWTI-mut1 decreased the serum lipase level, especially at doses of 2 mg/kg (590 U/L and 382 U/L, respectively, p<0.01) and 4 mg/kg (382 U/L and 235 U/L, respectively, p<0.01). However, there were no significant differences in the reduction of serum lipase levels between HWTI and HWTI-mut1 in all five groups.

**Figure 6:**
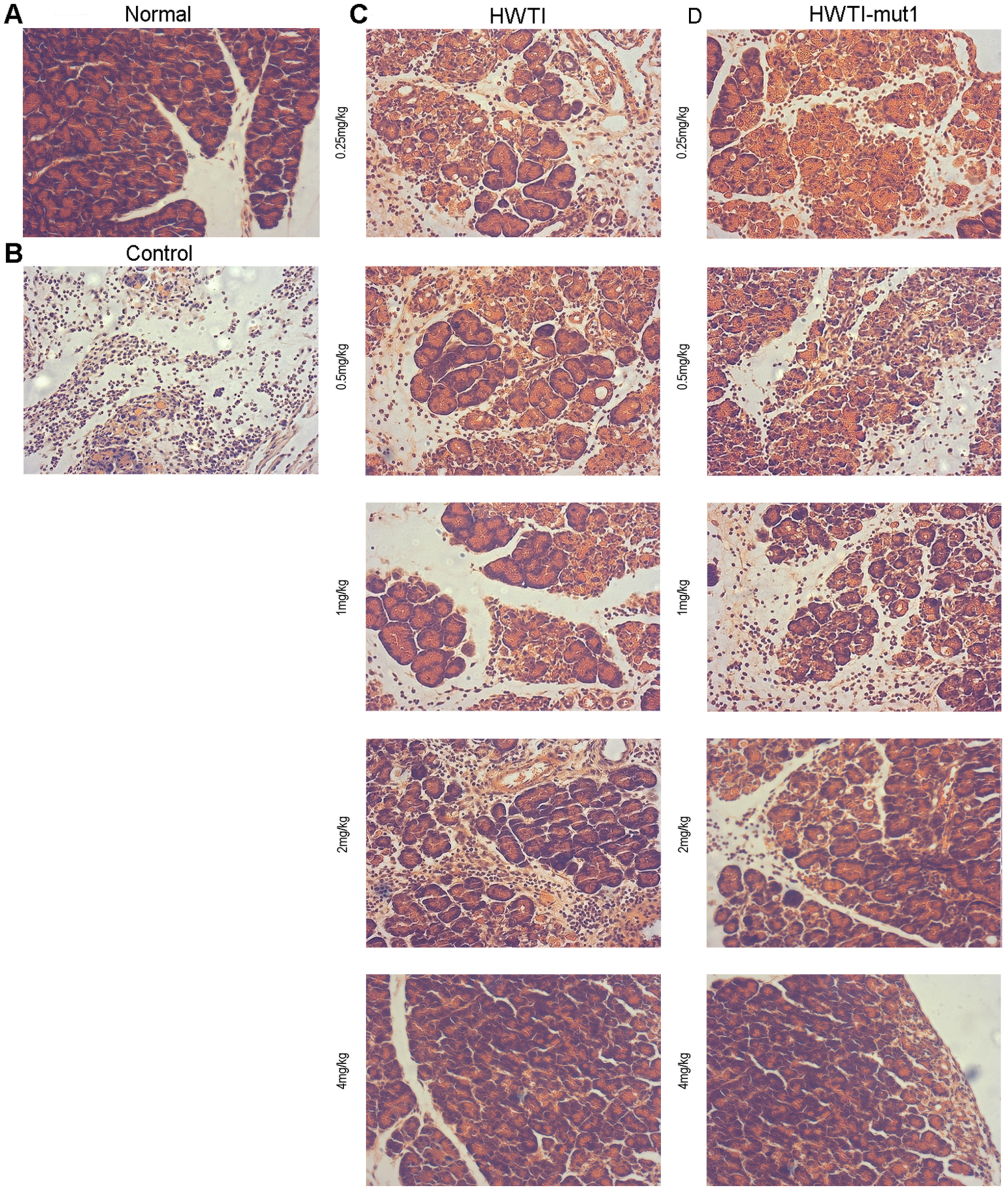
Protective effects of HWTI and HWTI-mut1 against damage to the pancreas. Pancreatic samples were obtained 72 hours after the induction of SAP. Formalin-fixed, paraffin-embedded sections of the pancreas were stained with hematoxylin and eosin. (A) Photomicrograph of the normal pancreas. (B) Mice were injected with saline after the induction of SAP. Sections show extensive acinar cell damage, edema and evident leukocyte infiltration. (C) Treatment with HWTI reduced the symptoms of acinar cell damage, leukocyte infiltration and edema. Normal acinar cell architecture was seen in the 4 mg/kg group. (D) Injection of HWTI-mut1 had a greater protective effect on the pancreas than HWTI. The 2 mg/kg and 4 mg/kg groups showed no significant damage to acinar cells. Magnification: 200×.

**Figure 7:**
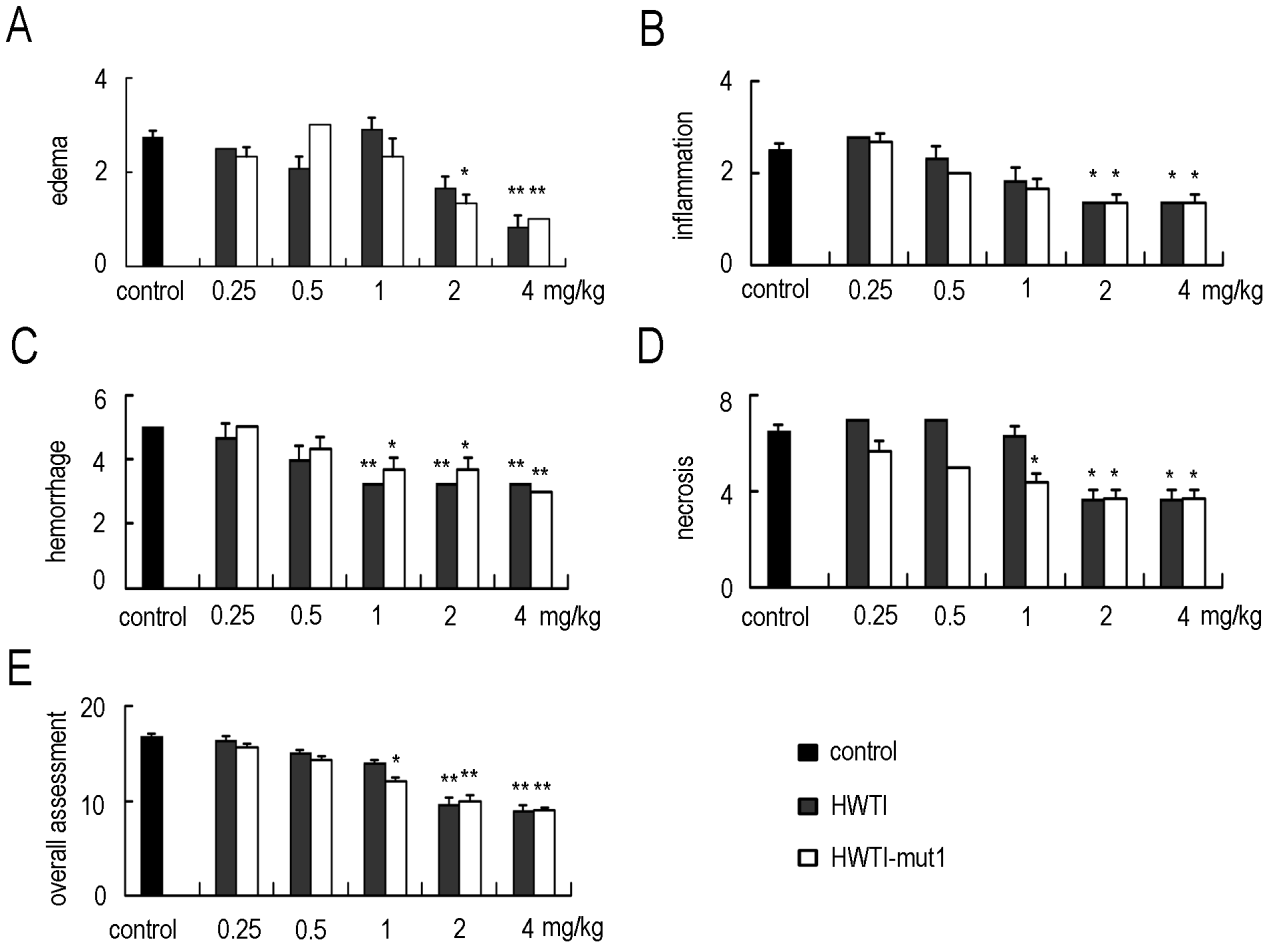
Histological scores. The scores include graded assessment of pancreas edema (A), inflammatory cell infiltration (B), hemorrhage (C), Score of necrosis (D). (E) Overall assessment of pancreas damage: sum of scores described above. Data are expressed as mean+SE (n = 3–5). * denotes p<0.05 compared with the control group; ** denotes p<0.01 compared with the control group.

Histological examination of sections of the pancreas confirmed the protective effect of the recombinant proteins against SAP (Fig. 6). Images of sections from the normal group show the typical architecture of the pancreas in the normal mouse (Fig. 6A). On the contrary, nearly all of the acinar cells were damaged and evident inflammatory cell infiltration was observed in the control group (Fig. 6B). Sections from mice treated with HWTI (Fig. 6C) and HWTI-mut1 (Fig. 6D) showed different histology depending on the treatment dose. Mice treated with low doses (lower than 1 mg/kg) showed severe acinar cell necrosis, prominent neutrophil infiltration and massive oedema and hemorrhage. These signs of acinar cell damage were significantly reduced with higher dose of 2 mg/kg, and at a dose of 4 mg/kg, no significant difference was detected between SAP and normal mice. Using the histological scoring method, pancreas edema, inflammatory cell infiltration, pancreas hemorrhage and acinar cell necrosis of sections of the pancreas were graded, respectively (Fig. 7A, B, C and D). Total histological scores were calculated and used to evaluate the overall tissue injury (Fig. 7E). In agreement with the observation above, the control group presented the highest total histological score of 9.75±1.50, indicating the most severe of SAP. However, the treatment of HWTI and HWTI-mut1 resulted in decreased histological scores significantly. In the mice treated with high doses (2 and 4 mg/kg), the scores for oedema, inflammatory cell infiltration, hemorrhage and necrosis were all reduced significantly, representing lower total histological scores (at 2 mg/kg, 9.66±0.66 for HWTI, 10.00±0.63 for HWTI-mut1; at 4 mg/kg, 8.95±0.62 for HWTI, 9.00±0.32 for HWTI-mut1) (Fig. 7E). There were no significant differences between HWTI and HWTI-mut1.

## Discussion

The present study aimed to assess the effect of mutations in HWTI on abolishing the potassium channel inhibitory activity of the protein and examine the therapeutic effect of HWTI and its mutant forms in mice with induced SAP. Two mutants, HWTI-mut1 and HWTI-mut2, were successfully designed and expressed in yeast. HWTI-mut1 could be expressed at a high level similar to HWTI. As expected, the potassium channel inhibitory activity was reduced in both mutants, which therefore showed lower neurotoxicity without impairment of their trypsin inhibitory function. Furthermore, HWTI-mut1 showed higher trypsin inhibitory activity than HWTI. Significantly, HWTI and HWTI-mut1 were effective in the treatment of mice in which SAP was induced by intraperitoneal injection of large doses of L-Arg. Both proteins effectively decreased the symptoms of SAP and prevented damage to acinar cells. Taken together, our results indicate that the potent inhibitory action and lower neurotoxicity of HWTI-mut1 make it an ideal candidate for the development of anti-acute pancreatitis drugs.

Extensive research efforts have been devoted to evaluating the effects of protease inhibitors in animal models of SAP. In most studies, inhibitors of trypsin and other related proteases exhibited a desirable protective effect when used before the onset of SAP. Tetsuya Hirano et al. evaluated the effect of the trypsin inhibitor urinastatin in a model of experimental acute pancreatitis induced by a supramaximal dose of cerulein. Urinastatin was administered 2 hours before, during or 1 hour after cerulein infusion. The authors reported that the administration of urinastatin before and during cerulein infusion may suppress the pathogenesis and evolution of pancreatitis [30]. In an acute pancreatitis model induced by pancreaticobiliary duct obstruction with cerulein stimulation and systemic hypotension in the rat, Tetsuya Hirano and Tadao Manabe further confirmed that preoperatively administered urinastatin was more protective than that used postoperatively [31]. More recently, in a study conducted by Yun Ju Jo et al., a human leukocyte elastase inhibitor, recombinant guamerin, significantly reduced the severity of cerulein-induced pancreatitis in rats when administered 30 min before the induction of pancreatitis [32]. In other studies, N-acetylcysteine (a strong antioxidant) and the cathepsin B inhibitor CA-074me administered preoperatively demonstrated beneficial effects in experimentally induced acute pancreatitis [33], [34]. Studies have shown that transient but high levels of trypsinogen activation are observed very early in most experimental models [35]. Therefore, treatment with protease inhibitors preoperatively can efficiently inhibit trypsinogen activation and reduce the severity of acute pancreatitis. This might explain why pretreatment can yield better protective effects as indicated above.

In clinical practice, pretreatment with protease inhibitors is actually impractical. Therefore, experts on pancreatitis in Japan recommend administering protease inhibitors as soon as the diagnosis of acute pancreatitis is confirmed [36]. In the present study, we evaluated the protective effect of the trypsin inhibitors (HWTI and HWTI-mut1) administered after the onset of SAP induced by L-arginine injection in mice. Administration of high doses of L-arginine, which is used as a relatively easy and reproducible model of pancreatitis, leads to significantly increased plasma amylase, pancreatic MPO activity, trypsin activation, and histological changes resembling acute pancreatitis in humans [27], [28]. Trypsin activity increased significantly after administration of L-arginine and continued increasing until 120 hours [28]. The prolonged increase in trypsin activity might contribute to the severity of the damage observed in this model, as serum amylase and lipase levels were increased significantly at 6 hours and reached a maximum at 72 hours, indicating that injury to the pancreas starts early and develops gradually [28]. This observation is in contrast to the transient pattern of trypsinogen activation in the cerulein-induced model of pancreatitis. More recently, Gaiser et al. developed an excellent mouse model of acute pancreatitis in which active trypsin can be conditionally expressed within pancreatic acinar cells using a tamoxifen-inducible genetic construct [1], [35]. In this model, intra-acinar trypsinogen activation was prolonged over a long time, which was also observed in the L-arginine-induced model. This study also clearly and elegantly established that intra-acinar trypsin can induce acute pancreatitis by itself when the intrinsic protective mechanisms are overloaded [1], [35]. These findings suggest that suppression of the prolonged trypsinogen activation by inhibitor treatment may decrease the severity of injury even after the onset of acute pancreatitis. In the present study, the trypsin inhibitors were administered intraperitoneally 8, 24 and 48 hours after the first injection of L-Arg and their effect was assessed after 72 hours. This not only provided adequate plasma levels of the inhibitors but also ensured that the treatment was administered after the diagnosis of acute pancreatitis was confirmed. Our results showed that HWTI and HWTI-mut1 were effective in the treatment of L-Arg induced acute pancreatitis in mice, which was likely due to their capacity to inhibit trypsin activity and therefore prevent damage to acinar cells.

It has been widely accepted that intra-acinar trypsinogen activation is sufficient to induce acute pancreatitis [1], [35]. However, increasing evidence indicates that trypsinogen activation is not the only factor determining the severity of pancreatitis. Compared with the original “trypsin paradigm”, the newer “multifaceted paradigm” has greatly broadened our understanding of acute pancreatitis. In the “multifaceted paradigm”, trypsin activity is one of several interrelated mechanisms that are simultaneously activated when acinar cells are injured [37]–[39]. Our results showing the beneficial effects of the recombinant trypsin inhibitors on L-arginine-induced acute pancreatitis raise the question of whether these agents can act by mechanisms other than trypsin inhibition and whether they can be effective to treat acute pancreatitis in other models. This issue warrants further study and may provide clues to further our understanding of the incidence, development and treatment of acute pancreatitis.
